# Asiatic Acid Induces Endoplasmic Reticulum Stress and Activates the Grp78/IRE1α/JNK and Calpain Pathways to Inhibit Tongue Cancer Growth

**DOI:** 10.3389/fphar.2021.690612

**Published:** 2021-05-26

**Authors:** Jialin Li, Kan Chen, Jianhua Huang, Dongqing Chu, Miaomiao Tian, Keqiang Huang, Chunyu Ma

**Affiliations:** ^1^ First Affiliated Hospital of Jinzhou Medical University, Jinzhou, China; ^2^ Graduated School of Jinzhou Medical University, Jinzhou, China; ^3^ Life Science Institute of Jinzhou Medical University, Jinzhou, China; ^4^ Second Affiliated Hospital of Jinzhou Medical University, Jinzhou, China

**Keywords:** asiatic acid, tongue cancer, endoplasmic reticulum stress, GRP78, calpain, apoptosis

## Abstract

Asiatic acid (AA) has been shown to induce apoptotic death in a range of cancers, but the mechanisms whereby it can inhibit tongue cancer growth have yet to be clarified. Herein, we explored the effects of AA on tongue cancer cells and found that it induced their apoptotic death *in vitro* and *in vivo,* while additionally impairing xenograft tumor growth *in vivo.* From a mechanistic perspective, AA treatment was associated with increases in levels of calcium and the calcium- dependent protease calpain, and it further induced endoplasmic reticulum (ER) stress and consequent Grp78-related IRE1α and JNK phosphorylation, ultimately driving caspase-3 activation and apoptotic death. Together, these results highlight AA as a promising tool for the therapeutic treatment of tongue cancer in clinical practice.

## Introduction

Tongue cancer is the most common form of oral cancer in the world and can manifest along the back, base, and edge of the tongue in affected individuals. This type of malignancy is associated with a poor prognosis owing to its high recurrence rates and tendency to exhibit aggressive growth (Xie et al., 2014; Taghavi and Yazdi, 2015), with approximately half of affected patients initially presenting with advanced disease. While a range of surgical, radiotherapeutic, and chemotherapeutic regimens have been formulated to treat tongue cancer, it still has the potential to recur or metastasize, with patients exhibiting a relatively persistent 5-year survival rate of 50% (Enomoto et al., 2018; Su et al., 2020). It is thus essential that novel therapeutic tools capable of treating tongue cancer be developed so as to improve patient outcomes.

Asiatic acid (AA) is a five-ring triterpenoid compound derived from *Centella asiatica* extracts. AA exhibits a range of pharmacological properties, including anti-inflammatory, antibacterial (Huang et al., 2011), antioxidant, cardioprotective, and neuroprotective activities (Nagoor Meeran et al., 2018). AA treatment has been shown to suppress the growth of breast (Hsu et al., 2004) and lungcancer cells (Wu et al., 2017), without inducing substantial toxicity in non-malignant cells (Siddique et al., 2017). As our preliminary studies have suggested that AA is additionally capable of inhibitingtongue cancer cell growth, we conducted the present study in an effort to better understand the mechanistic basis for the anti-cancer activity of this promising therapeutic compound.

The endoplasmic reticulum (ER) represents a key site of cellular protein, lipid, and carbohydrate biosynthesis, and it additionally sequesters intracellular calcium ions and controls their availability (Iurlaro and Muñoz-Pinedo, 2015). The disruption of ER homeostasis under a range of pathophysiological conditions can induce substantial ER stress, whereupon the unfolded protein response (UPR) is initiated in an effort to alleviate such stress and to restore homeostaticnormalcy. The ER stress response serves as an important mediator of cell survival or apoptosis under specific conditions (Schwarze et al., 2008). Briefly, low-level induction of this ER stress response can enhance cell survival under adverse conditions, whereas high-intensity or prolonged UPR induction can instead induce apoptotic cell death associated with a failure to restore homeostasis via the calpain (Cubillos-Ruiz et al., 2017) and IRE1α/ JNK signaling pathways (Urano et al., 2000). However, excessive ER stress can lead to a breakdown of cell homeostasis, thereby triggering apoptotic cell death. There are three ER-resident sensor proteins - IRE1α, PERK, and ATF6. These sensors transduce information regarding the protein folding status of the ER to the cytosol and nucleus to restore the protein folding capacity. Under ER stress conditions, these sensors are activated by BiP dissociation and/or direct misfolded protein binding. Activated IRE1α can activate the JNK, p38, ERK, and NF-kB pathways, thus modulating diverse cellular pathways in an XBP1-independent manner. Activated PERK can activate the downstream protein p-eIF2α, in turn inducing the upregulation of CHOP, which plays an important role in promoting cell apoptosis. ATF6 is transported to the Golgi apparatus under ER stress conditions, where it is processed by S1P and S2P, releasing its cytosolic domain fragment as a transcription factor. ATF6 activates genes encoding protein chaperones, ERAD components, and XBP1. In addition, ER stress is usually accompanied by intracellular calcium overload. Calpain is a protease that participates in altering calcium content levels, with rising calcium levels having the potential to promote calpain activation. Activated calpain can cleave pro-caspase-12 to caspase-12, thus triggering ER stress-mediated apoptosis.

Many recent studies have found that ER stress induction-related apoptosis is closely tied to the pathogenesis of a range of cancers. Asiatic acid also induces glioblastoma multiforme cell apoptosis via ER stress(Kavitha et al., 2015). As such, we herein explored the impact of AA treatment on tongue cancer growth *in vitro* and *in vivo*, and explored the association between such activity and ER stress induction.

## Materials and methods

### Cell line and reagents

AA was obtained from the National Institute for Food and Drug Control (Nanjing Jingzhu, China). Antibodies specific for calpain, cleaved caspase-3, JNK, p-JNK, Grp78, Bax, and Bcl-2, and actin were from Cell Signaling Technology (Shanghai, China), while Beyotime Biotechnology (Shanghai, China) was the source of BCA kits, Fluo-4AM fluorescent dye, SDS-PAGE gel preparation kits, secondary HRP goat anti-rabbit IgG, HRP-goat anti-mouse IgG, anti-IRE1α, and anti-P-IRE1α. The human Tca8113 tongue cancer cell line was obtained from the Cell Bank of the Chinese Academy of Science (Shanghai, China).

### MTT assay

Tca8113 cells were seeded in 96-well plates (8×10^4^/well) and allowed to adhere, after which cells were treated with AA (0, 10, 20, 30, 40, 50, 80, or 100°μM) for 24°h, after which MTT reagent was added to each well (20°μL/well) for 4°h at 37ºC. Media was then removed from each well and replaced with 150°μL of DMSO. After 10°min of constant agitation, absorbance at 570°nm in each well was assessed via microplate reader.

### Colony Formation Assay

Tca8113 cells were added to 6-well plates (5×10^2^/well) for 24°h, after which AA (40°μM) was added. Following a 1-week treatment period, colonies were stained using 0.5% crystal violet and imaged.

### Calcium Ion Detection

Tca8113 cells were added to 24-well plates (6×10^4^/well) for 24°h, after which they were treated for 6° h with AA (40°μM). Next, each well was treated with Fluo-4AM (1°μM) for 20°min. Cells werethen washed with PBS, incubated for 25°min to facilitate Fluo-4AM conversion to Fluo-4, and imaged via fluorescent microscopy.

### Immunofluorescent staining

When cells were 80% confluent, they were rinsed in PBS, fixed for 15°min with 4% paraformaldehyde (PFA), permeabilized for 20°min with Triton X-100 (0.5%), washed thrice with PBS, and blocked for 30°min with 2% BSA. They were then probed overnight with anti-Grp78 (1:250) at 4°C, after which they were probed with appropriate fluorescently-conjugated secondary antibodies for 1°h at room temperature. DAPI was then applied for 5°min to counterstain nuclei, after which cells were imaged by confocal microscope.

### Assessment of Cell Apoptosis

Tca8113 cells in 6-well plates were treated with AA (40°μM) for 24°h, after which cells were stained with Hoechst 33342, after which apoptotic cells were observed via fluorescence microscope (Olympus, Tokyo, Japan). In addition, cells were stained with Annexin V and PI to confirm differences in apoptotic death among treatment groups. Briefly, following a 12°h AA treatment (40°μM), cells were harvested, rinsed, centrifuged at 2000°rpm for 5°min, and resuspended in 400°μL binding buffer containing 5°μL Annexin V-FITC and 10°μL propidium iodide (PI) for 10°min. Cells were then evaluated via flow cytometry within 1°h to establish the percentages of cells in theearly and late stages of apoptotic death.

### Western Blotting

Following A treatment, cells were lysed with a buffer containing protease inhibitors (Roche). Equal amounts of protein from each sample (40°μg) were then separated via SDS-PAGE and transferred to PVDF membranes, which were blocked for 1°h using 2% BSA prior to being probed overnight with appropriate primary antibodies (1:1000) at 4°C, followed by probing for 1°h with secondary HRP- conjugated anti-mouse or anti-rabbit IgG (Cell Signaling, Shanghai, China). An enhanced chemiluminescence ECL Plus system (Beyotime Institute of Biotechnology, Shanghai, China) was then used to detect protein bands, with band densitometry subsequently being analyzed with a scanning densitometer (Bio-Rad) and associated analytical software.

### Animals

Male BALB/cANNCjr nu/nu mice (20-25°g) were obtained from Beijing Vital River Laboratory Animal Technology Co., Ltd., Beijing, China and were housed in a climate-controlled facility (22- 24°C, 12°h light/dark cycle) with free food and water access. Animals were allowed to acclimate to laboratory conditions for at least 1°week prior to experimental use. All animal studies were approved by the Experimental Animal Ethics Committee of Jinzhou Medical University, and were performed in a manner consistent with the NIH Guide for the Care and Use of Laboratory Animals published (Publication, 8^th^ Edition, 2011).

### Xenograft tumor models

Mice (8-weeks-old) were subcutaneously implanted with 2×10^6^ Tca8113 cells in the flank region. Once tumors had grown to a volume of ∼100°mm^3^, animals were intraperitoneally injected once per day with AA in 0.1% DMSO (15°mg/kg/d) or with an equivalent volume of 0.1% DMSO. Vernier calipers were used to assess tumor volume as follows: length × width^2^/2. Following a 4-week monitoring period, mice were euthanized via pentobarbital sodium injection (75°mg/kg), at which time tumors were collected, weighed, and imaged.

### TUNEL Staining

On day 28 post-AA treatment, mice were euthanized and tumor tissue samples were collected, paraffinized, and cut to yield 4-μm-thick sections. Apoptotic death in these sections was evaluated using an In situ Cell Death Detection Kit (Roche, IN, United States) based on provided directions. The TUNEL staining of these sections was first performed, after which DAB was applied to detect the labeled apoptotic cells, and hematoxylin was applied for nuclear counterstaining. Numbers of TUNEL-positive nuclei in five random fields of view from each tissue section were assessed in a blinded fashion, with the results being expressed as a fraction of the total nuclei visible in a given field.

### Statistical Analysis

Data are means ± SD, and were compared via one-way ANOVAs with Bonferroni/Dunn tests. *p* < 0.05 was the significance threshold for this study.

## Results

### AA Inhibits Tongue Cancer Cell Viability and Proliferation

**FIGURE 1:**
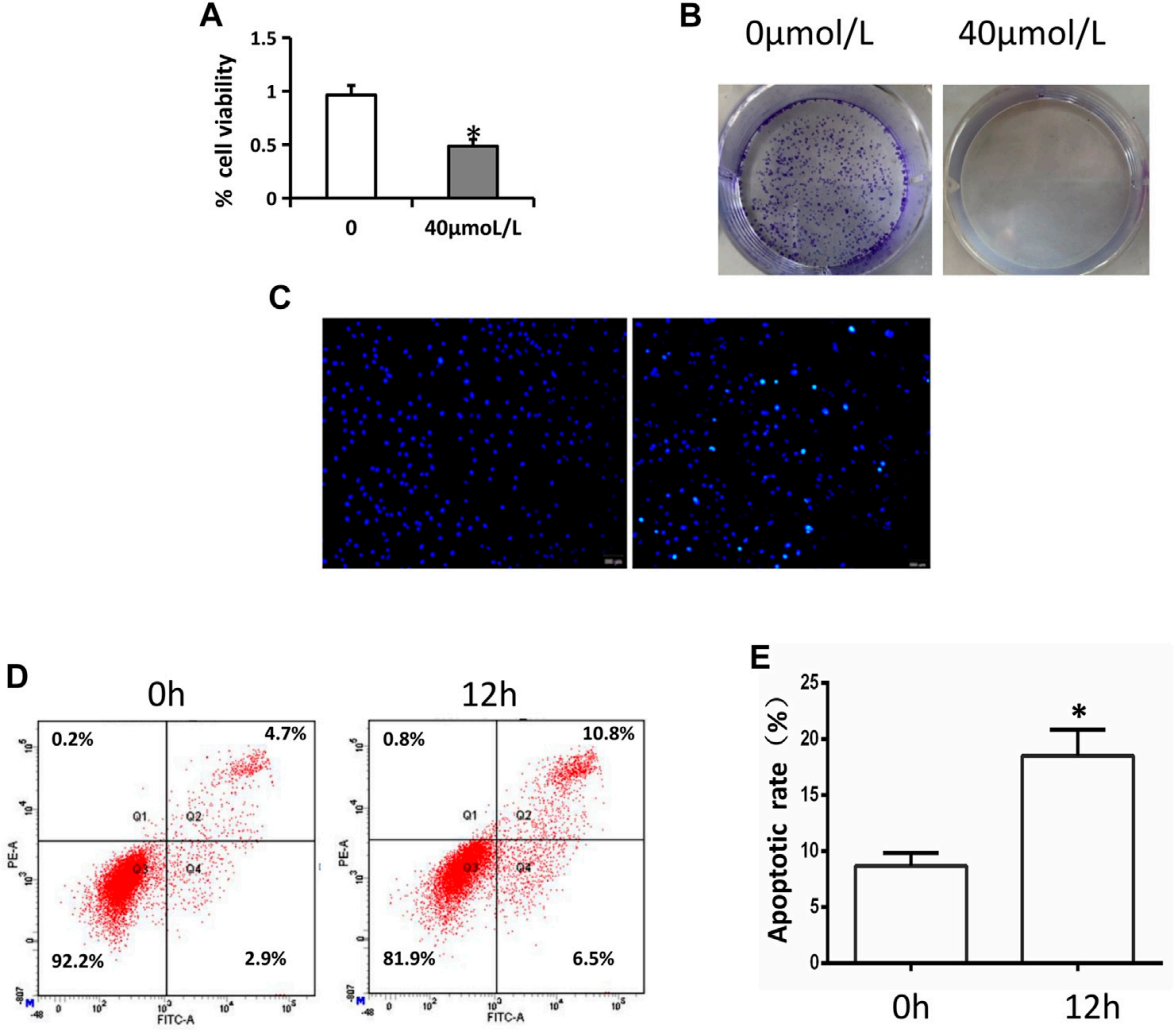
AA inhibits tongue cancer cell viability and proliferation. **(A)** AA treatment (40°μM) inhibited Tca8113 cell viability as measured via MTT assay. *p < 0.01 vs. untreated. **(B)** AA (40°μM) suppressed Tca8133 cell colony formation activity over a 6-day period. **(C)** AA treatment (40°μM) induced the apoptotic death of Tca8113 cells as measured via Hochest 33342 staining. Apoptotic nuclei are marked with arrows. **(D)** AA treatment (40°μM) induced the apoptotic death of Tca8113. cells as measured via flow cytometry. *p<0.01 vs. 0°h.

We began by assessing the impact of AA on tongue cancer cell viability via an MTT assay, which revealed that AA application significantly suppressed the viability of these cells with an IC50 value of approximately 40°μM (Figure 1A). Consistent with this, AA treatment (40°μM) significantly suppressed the colony forming activity of Tca8113 cells (Figure 1B).

### AA Induces Tongue Cancer Cell Apoptotic Death

The apoptotic death of these tongue cancer cells was next evaluated via Hoechst 33342 stainingand flow cytometry. In Hoechst 33342 staining analyses, AA was found to cause the cells to shrinkand the fluorescence to increase, consistent with the increased apoptosis of Tca8113 cells relative to control treatment (Figure 1C), and this was confirmed via flow cytometry (Figure 1D, E).

### AA Treatment Alters Apoptosis-Related Protein Levels In Tongue Cancer Cells

**FIGURE 2:**
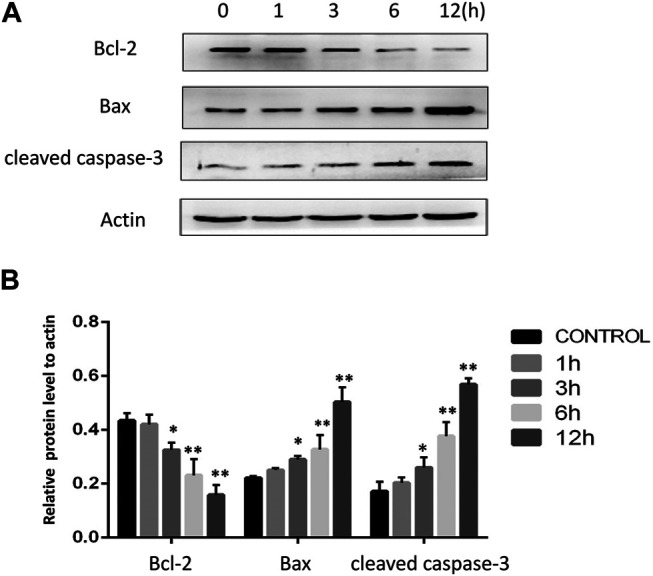
AA treatment reduces Bcl-2 expression and enhances cleaved caspase-3 and Bax levels in tongue cancer cells. **(A)** The impacts of AA treatment (40°μM) on Bcl-2, Bax, and cleaved caspase-3 were assessed via Western blotting. **(B)** AA treatment (40°μM) for 3°h significantly reduced Bcl-2. expression and increased cleaved caspase-3 and Bax levels. *p<0.05 vs. 0°h, **p<0.01 vs. 0°h.

To confirm the induction apoptosis in tongue cancer cells following AA treatment, mitochondrial apoptotic pathway related-protein levels were next assessed via Western blotting. AA treatmentwas associated with significant reductions in the levels of anti-apoptotic Bcl-2 and with increases inpro-apoptotic Bax and cleaved caspase-3 levels (Figure 2B). This suggests that AA can induce Tca8113 cell apoptosis via the mitochondrial pathway.

### AA Treatment Increases Calcium Ion Levels and Calpain Expression in Tongue Cancer Cells

**FIGURE 3:**
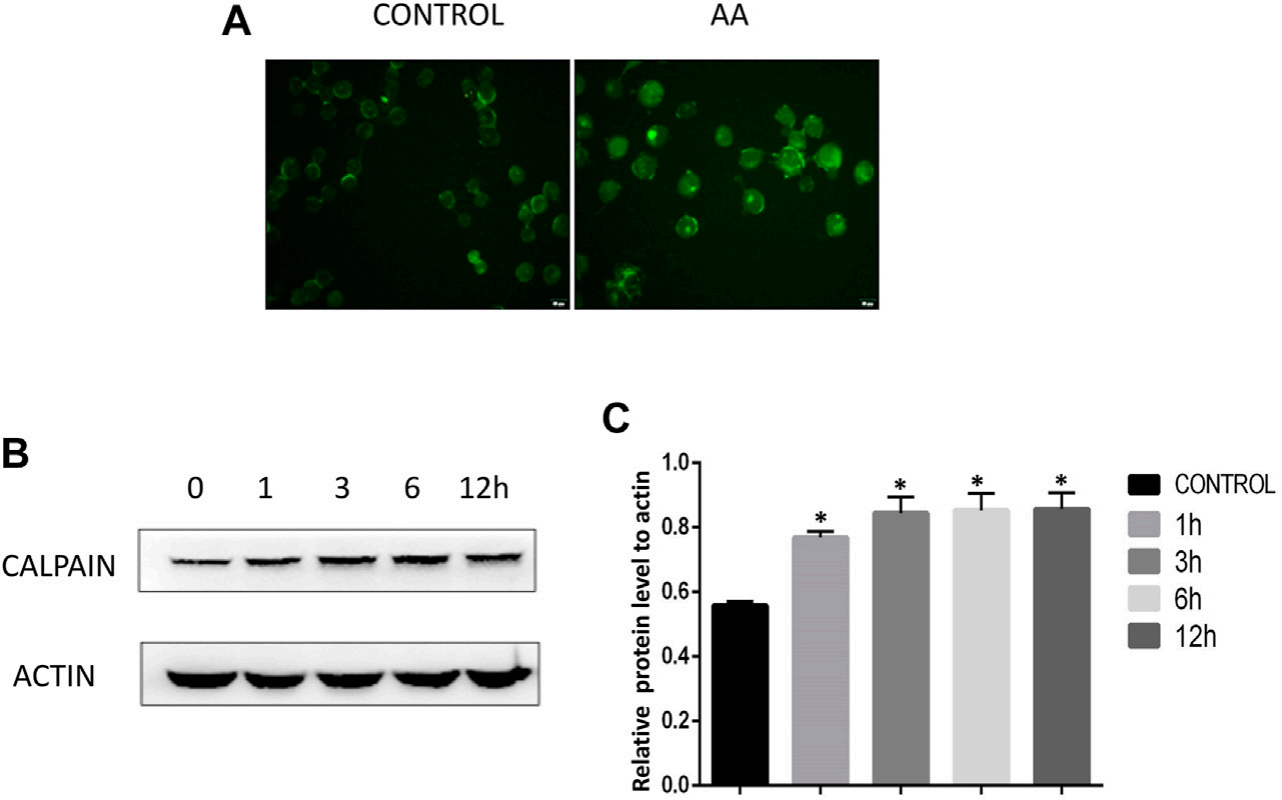
AA treatment increases calcium ion levels and calpain expression in tongue cancer cells. **(A)** Fluo-4AM staining revealed significant increases in calcium ion levels in tongue cancer cells following AA treatment. **(B)** Western blotting revealed significant increases in calpain protein levels in tongue cancer cells following a 1°h treatment with AA relative to baseline levels. *p<0.01 vs. 0°h.

Calcium and the calcium-dependent protease calpain are important regulators of apoptotic death. We therefore used the Fluo-4AM probe to assess calcium levels in Tca8113 cells, revealing that AA treatment (40°μM) for 6°h was associated with a significant increase in intracellular calcium ion levels relative to control treatment (Figure 3A). Western blotting further revealed that calpain levels rose in a time-dependent fashion following AA treatment (Figure 3B, C), suggesting that AAcan increase intracellular calcium levels and calpain expression in tongue cancer cells, thereby driving their apoptotic death.

### AA Treatment Promotes the Activation of the Grp78/ Ire1α/Jnk Pathway

**FIGURE 4:**
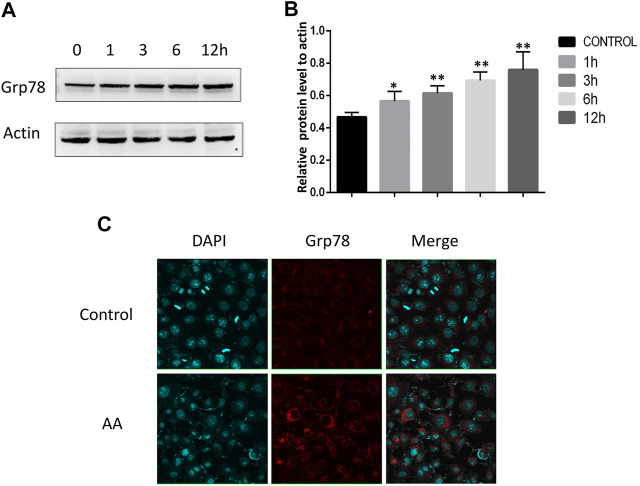
AA increases Grp78 expression in tongue cancer cells. **(A)** Western blotting was used to assess the impact of AA treatment (40°μM) on Grp78 levels after 0, 1, 3, 6, and 12°h. **(B)** AA treatment significantly increased Grp78 protein levels after 1°h relative to baseline. *p<0.05 vs. 0°h, **p<0.01 vs. 0°h. **(C)** Immunofluorescent staining of Grp78 levels in tongue cancer cells revealed. significant increases in these levels upon AA treatment. Experiments were repeated in triplicate.

**FIGURE 5:**
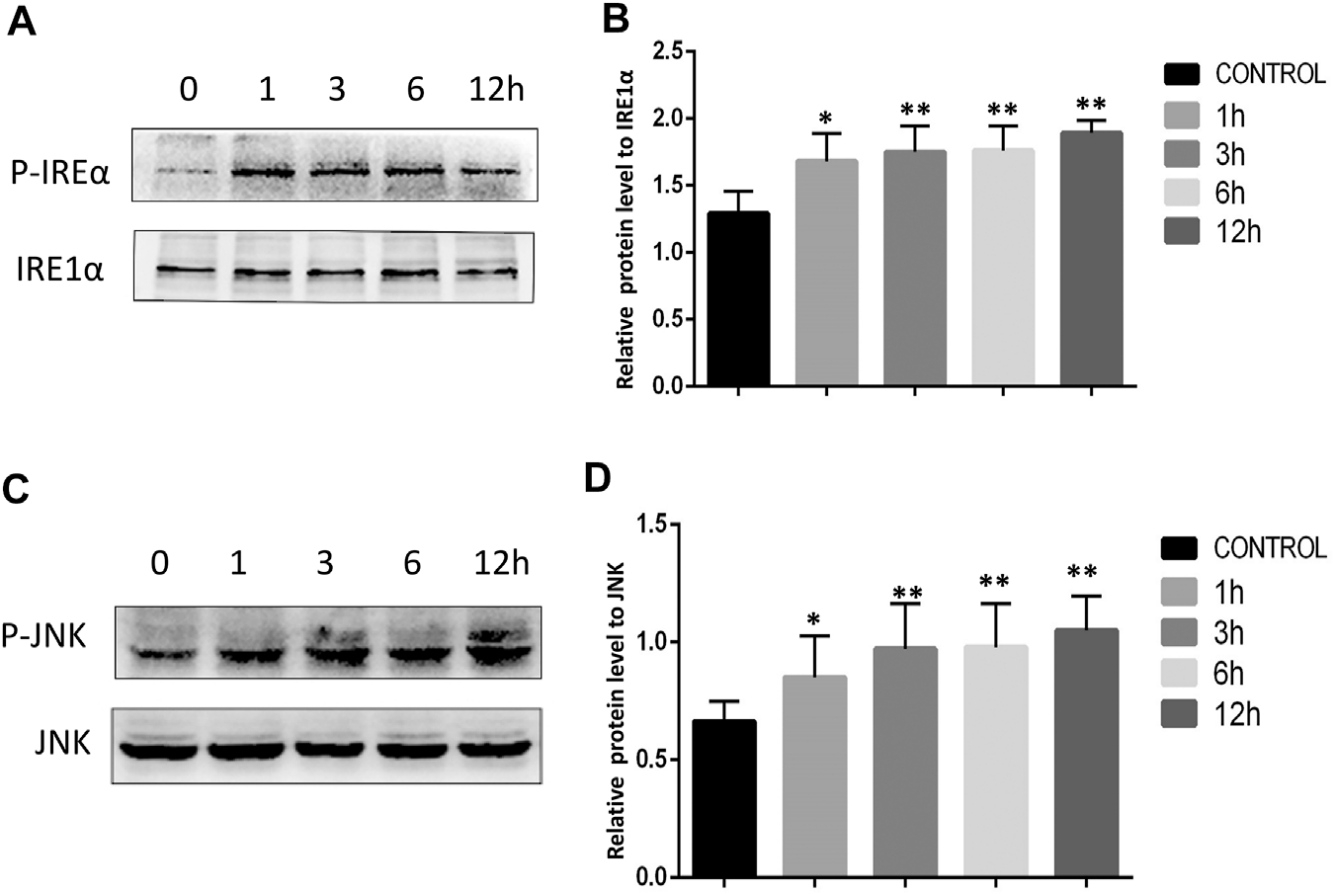
AA promoted increased IRE1α and JNK phosphorylation. **(A)** Western blotting was used to assess the impact of AA on IRE1α phosphorylation after 0, 1, 3, 6, and 12°h. **(B)** AA treatment was associated with significantly increased IRE1α phosphorylation following a 1°h treatment relative to control. *p<0.05 vs. 0°h, **p<0.01 vs. 0°h. **(C)** Western blotting was used to assess the impact of AA on JNK phosphorylation after 0, 1, 3, 6, and 12°h. **(D)** AA treatment was associated with significantly. increased JNK phosphorylation following a 1°h treatment relative to control. *p<0.05 vs. 0°h, 369 **p<0.01 vs. 0°h.

The ER stress marker protein Grp78 and downstream IRE1α/JNK signaling are closely linked to the induction of apoptotic cell death under adverse conditions. We therefore assessed Grp78 levels in Tca8113 cells via immunofluorescent staining and Western blotting, revealing significant increases in Grp78 levels following AA treatment (Figure 4A-C). The activation of IRE1α and JNK was further assessed via Western blotting, revealing that AA treatment significantly increased P-IRE1α and P-JNK levels in these tongue cancer cells in a time-dependent fashion without increasing overall IRE1α and JNK protein levels (Figure 5A-D), indicating that AA can activate the Grp78 IRE1α/JNK pathway

### AA Suppresses Tongue Cancer Tumor Growth in Vivo by Inducing Apoptotic Tumor Cell Death

**FIGURE 6:**
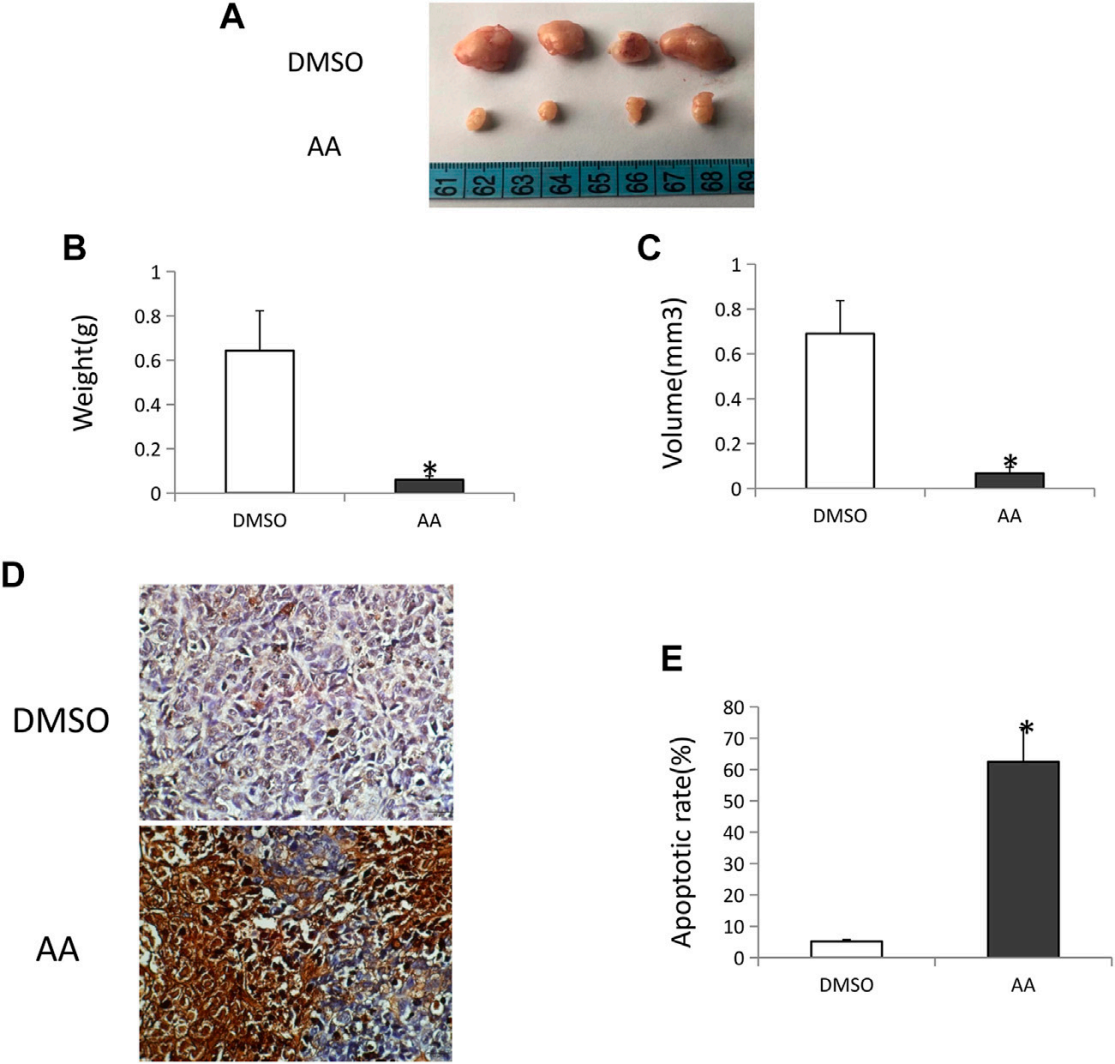
AA inhibits tongue cancer growth and induces tumor cell apoptotic death *in vivo.* **(A)** Tongue cancer growth in xenograft model nude mice following AA or DMSO vehicle control treatment (*n* = 4/group). **(B)** AA treatment was associated with significant reductions in tumor weight relative to DMSO control. *p <0.01. **(C)** AA treatment was associated with significant reductions in tumor volume relative to DMSO control. *p <0.01. **(D)** TUNEL staining of vehicle **(A)** and AA **(B)** treated tongue cancer samples. **(E)** AA treatment induced significantly more tongue cancer cell apoptosis relative to DMSO treatment. *p<0.01.

Nude mice were next implanted with Tca8113 tumors to evaluated the *in vivo* impact of AA treatment of tongue cancer tumor growth. This analysis revealed AA treatment to be associated with significant reductions in tumor volume and tumor weight relative to control treatment (Figure 6A-C). TUNEL staining of collected tumor tissue sections revealed that there were significantly more apoptotic tumor cells in mice treated with AA relative to control mice (Figure 6D, E), suggesting that AA suppresses tongue cancer growth by inducing the apoptotic death of these tumor cells.

## Data Availability

The raw data supporting the conclusions of this article will be made available by the authors, without undue reservation, to any qualified researcher.

## Discussion

Several studies have shown that Asiatic acid exhibits antiproliferative effects when used to treat various tumor cell types including hepatoma (HepG2)(Lu et al., 2016), ovarian cancer (SKOV3 and OVCAR-3)(Ren et al., 2016), and Cholangiocarcinoma (KKU-156 and KKU-213) cells(Sakonsinsiri et al., 2018). Despite its robust antitumor properties, no prior studies have yet to define the mechanism whereby AA can suppress tongue cancer cell growth. Herein, we began by exploring the impact of AA on tongue cancer cell viability, revealing that it significantly impaired tongue cancer cell growth and induced the apoptotic death of these cells in a time-dependent fashion.Similarly, AA treatment of mice bearing xenograft tumors was sufficient to impair tumor growth and to increase the frequency of apoptotic tumor cells, suggesting that AA can inhibit tongue cancer by driving these tumor cells to undergo apoptosis.

The two primary signaling mechanisms that induce apoptotic cell death are the mitochondrial and the death receptor signaling pathways. Bcl-2 family proteins include Bcl-2, which inhibits mitochondrial apoptotic signaling pathway activation, and Bax, which counteracts Bcl-2 activity in cells (Ashkenazi et al., 2017). When apoptotic signaling cascades are initiated in cells, Bax and other Bcl-2family proteins rapidly localize to the mitochondrial membrane where they can form membrane poreswhich facilitate the release of mitochondrial cytochrome c into the cytoplasm (Germain et al., 2002). To assess the mechanism whereby AA administration promoted tongue cancer cell apoptosis, we assessed Bcl-2, Bax, and cleaved caspase-3 levels in treated cells. These experiments revealed that AA induced mitochondrial apoptotic pathway activation, as evidenced by Bcl-2 downregulation and upregulation of both Bax and cleaved caspase-3 levels following treatment.

Apoptotic signaling can be regulated by a range of mechanisms, including the ER stress response. The release of calcium ions from the ER into the cytoplasm is a hallmark of such ER stress, and calpain upregulation has previously been linked to apoptosis and the activation of caspases including caspase-12 and caspase-3 in the context of disrupted calcium homeostasis (Bahar et al., 2016; Zhang et al., 2017). Herein, we found that AA treatment was associated with a significant increase in intracellular calcium ion levels and calpain expression in tongue cancer cells. As such, AA-induced apoptosis is likely at least partially associated with this calcium-mediated calpain pathway. The ER stress marker protein Grp78 and associated IRE1α /JNK signaling can also regulate ER stress- induced apoptosis. The type I transmembrane protein IRE1α exhibits a cytoplasmic serine/threonine kinase domain (Hetz and Papa, 2018). In normal physiological contexts, IRE1α binds to the ER chaperone Grp78. When cells are faced with ER stress, however, the accumulation of unfolded proteins results in Grp78 dissociation from IRE1α, which enables this receptor to interact with TRAF2 and ASK1, generating the IRE1α-TRAF2-ASK1 complex, that can activate JNK signaling (Nishitoh et al., 2002; Hu et al., 2006; Sano and Reed, 2013), thereby triggering mitochondrial apoptosis pathway activation via suppressing Bcl-2 expression and enhancing Bax expression (Yamamoto et al., 1999; Bassik et al., 2004; Puthalakath et al., 2007). This, in turn, results in the release of cytochrome C into the cytosol wherein it can bind to caspase-9, activate caspase-3, and thereby drive apoptotic cell death. Overall, we determined that AA treatment resulted in an increase in Grp78 expression and associated enhancement of IRE1α and JNK phosphorylation in tongue cancer cells, indicating that AA induces the apoptotic death of these tumor cells via the activation of the ER Grp78/IRE1α/JNK signaling pathway.Although we found that AA induced ER stress and activated the Grp78/IRE1α/JNK and Calpain pathways to inhibit tongue cancer growth. However, there are certain limitations to this study. CHOP and caspase-12 play an important role in the ER stress induced-cell death (Cheng and Dong, 2018). As such, it is important that the roles of CHOP and caspase-12 in AA-induced tongue cancer cell death be confirmed. Furthermore, a reticular stress inhibitor compound such as salubrinal should be used to verify that AA induces ER stress in tongue cancer cells. These topics will be a focus for our future research.

In conclusion, we found that AA exhibits effective antitumor properties, suppressing Tca8113 tongue cancer cell growth and activating proteins related to the IRE1α/JNK signaling pathways under conditions of ER stress, suggesting that this may be one mechanism whereby Asiatic acid induces apoptosis. Owing to the complex, multi-level regulation of ER stress responses, however, further research will be essential to fully elucidate the mechanisms whereby AA induces and modulates ER stress responses in these cancer cells. Even so, our data suggest that AA holds great promise for the treatment of tongue cancer, and future studies of the underlying pathways thus have the potential to identify novel therapeutic avenues to the treatment of this deadly disease.
